# Joint Design of a Simultaneous Reflection and Transmission RIS in Mode-Switching Mode to Assist NOMA Systems

**DOI:** 10.3390/s23125504

**Published:** 2023-06-11

**Authors:** Xiaoping Zhou, Hanqi Wang, Jiajia Chen

**Affiliations:** The College of Information, Mechanical and Electrical Engineering, Shanghai Normal University, Shanghai 200234, China; hanqi_77@163.com

**Keywords:** reconfigurable intelligent surfaces, non-orthogonal multiple access, beamforming, simultaneous transmission and reflection

## Abstract

Simultaneous transmitting and reflecting reconfigurable intelligent surfaces (STAR-RISs) can reflect signals and transmissive signals simultaneously and can extend the coverage of signals. A conventional RIS mainly focuses on the case where the signal source and the target are on the same side. In this paper, a STAR-RIS-assisted non-orthogonal multiple access (NOMA) downlink communication system is considered to maximize the achievable rate for users by jointly optimizing the power-allocation coefficients, active beamforming, and STAR-RIS beamforming under the mode-switching (MS) protocol. The critical information of the channel is first extracted using the Uniform Manifold Approximation and Projection (UMAP) method. Based on the key extracted channel features, STAR-RIS elements and users are clustered individually using the fuzzy C-mean clustering (FCM) method. The alternating optimization method decomposes the original optimization problem into three sub-optimization problems. Finally, the sub-problems are converted to unconstrained optimization methods using penalty functions for the solution. Simulation results show that when the number of elements of RIS is 60, the achievable rate of the STAR-RIS–NOMA system is about 18% higher than that of the RIS–NOMA system.

## 1. Introduction

With the rapid development of modern mobile communication technologies, the capacity of communication networks will grow significantly in the next decade, and the high complexity of networks, the high cost of hardware, and the increasing consumption of resources have become the key issues facing wireless communication in the future [1]. Reconfigurable intelligent surfaces (RISs) are low-cost, programmable, easy to deploy, and low-energy, which makes RISs stand out in competition with other technologies. RISs and their variants [2,3] have become promising technologies for sixth-generation (6G) wireless networks. Simultaneous transmitting and reflecting (STAR)-RIS is a new technique in RISs; whereas a conventional RIS focuses on the case where the signal source and the target are located on the same side of the RIS [4,5,6], a STAR-RIS is capable of simultaneous transmission and reflection, and the target can be distributed on both sides of the signal source, allowing for full spatial coverage and enabling better-aided system communication [7,8,9].

Due to the significant capacity growth of future communication networks, non-orthogonal multiple access (NOMA) [10,11,12,13,14] has also received considerable attention as a promising technology to enhance spectrum efficiency and support large-scale connectivity. NOMA transmits the information of multiple users on the same sub-channel. Interference information is introduced, in contrast to orthogonal multiple access (OMA) systems, where each user has a separate resource in the time, frequency, or code domain [15]. NOMA works through sharing time-frequency resources by separating users into other domains [16]. Power-domain-based NOMA maximizes performance by assigning different power levels to users. The receiver-side detection process of power-domain NOMA highly depends on successive interference cancellation (SIC) [17]. The SIC receiver performs interference cancellation in a specific order according to different users’ signal power levels and distinguishes between other users [18]. The main advantages of NOMA are: (1) NOMA’s resource allocation method is based on a non-orthogonal, and this approach can improve spectrum efficiency. (2) NOMA uses a scheduling-free access scheme, which can better meet latency and reliability requirements. (3) NOMA can be more easily combined with other coding schemes. Combining RIS with NOMA can provide services to more users and result in higher network performance [19,20].

In the study of assisted communication, the design of the beamforming is a major concern for researchers. Ref. [21] considered joint beamforming for RIS-assisted hybrid satellite–terrestrial relay networks to minimize the total transmit power of satellites and base stations (BSs) while ensuring user rate requirements. In [22], a confidential and energy-efficient hybrid beamforming scheme for a satellite-ground integrated network was studied, and a hybrid beamformer for the base station and a digital beamformer for the satellite was jointly designed. The problem of maximizing spectral efficiency was considered in [23] for RIS-assisted single-user communication systems. In [24], the authors applied active RISs to collaboratively enhance secure transmission from the base station to the mobile user to maximize the achievable secrecy rate subject to transmit power constraints and interference thresholds. The authors in [25] used RISs to augment artificial noise to assist multiple-input multiple-output (MIMO) secure communication systems. In [26], a Compressed Sensing (CS)-based channel estimation method was proposed by exploiting the sparse representation of cascaded channels. In [27], a globally optimal solution for a single-user system’s transmit-power-minimization problem was obtained using a branch-and-bound approach. The authors of [28,29] used semidefinite relaxation and streamlined optimization, respectively, to maximize the received signal-to-noise ratio of the multiple-input single-output (MISO) communication systems for RISs. Ref. [30] solved the RIS-assisted NOMA downlink-reachability and rate-maximization problem using semi-positive definite planning (SDP). Ref. [31] derived uplink-outage probability expressions for blind RIS–NOMA using RIS as an innovative reflector and RIS as an access point (AP). The results showed that adding RIS elements enhanced the total capacity and interruption performance. Some researchers have also conducted in-depth studies on STAR-RIS-assisted communication. In [32], the authors studied STAR-RIS-assisted dual-user communication systems and proposed the problem of maximizing the total coverage of NOMA and OMA systems. In [33], the power-minimization problem for STAR-RIS-assisted unicast and multicast systems was studied for different operating protocols of STAR-RIS, and the active-passive beamforming was jointly optimized. In [34], the authors proposed the index modulation (IM)-assisted STAR-RIS–NOMA system to improve spectral efficiency. In [35], the authors considered the STAR-RIS-assisted full-duplex (FD) wireless communication system. They derived analytical closed-form expressions for the outage probability and achievable throughput for uplink and downlink communication. Ref. [36] studied the STAR-RIS-assisted NOMA downlink communication system, maximizing the signal-to-noise ratio (SNR) to design the reflected and transmitted phase-shift matrices. In [37], the power of the STAR-RIS-enabled uplink NOMA system was minimized by a joint optimization scheme. The authors of [38] investigated the energy-saving potential of STAR-RIS in a MIMO-enabled NOMA system. However, the studies mentioned above on STAR-RIS are all processed directly on the high-dimensional channel, which is challenging to solve.

Based on the above background, integrating STAR-RIS with the NOMA system is of great practical importance. The main contributions of this paper are summarized as follows.

In the STAR-RIS–NOMA system, we jointly optimize the user’s power-allocation coefficient, active beamforming at the BS, and passive beamforming at the STAR-RIS to maximize the achievable sum rate. Since the number of antennas at the BS and the number of STAR-RIS elements are large, and thus the associated channel dimension is high, we use Uniform Streaming Approximation and Projection (UMAP) to extract the key features of the channel and reduce the complexity of the solution. The elements of the STAR-RIS are clustered to improve the efficiency of the whole communication system.

We decouple the original problem into three sub-problems for solving a given decoding order. Regarding the optimization problem of the power-allocation coefficients, we use SCA with a penalty function to solve it. After adopting the SDR relaxation constraint, the block coordinate descent (BCD) method is used to solve the active beamforming at the BS and the passive beamforming at the STAR-RIS.

Simulation results show: (1) combining the STAR-RIS with NOMA can significantly improve the system’s performance, and the system performance increases with the number of STAR-RIS elements; (2) the performance of the STAR-RIS–NOMA system is better than that of the reflection/transmission RIS–NOMA-only system.

The rest of the paper is organized as follows: Section 2 presents the STAR-RIS–NOMA system’s communication model and problem representation. Section 3 performs feature extraction of the channel and clusters the users and STAR-RIS elements. A new algorithm for alternating optimization is proposed in Section 4 to solve the joint optimization problem. Numerical results are given in Section 5. Section 6 concludes the whole paper.

Notations: Scalars, vectors and matrices are represented in lowercase, bold lowercase and uppercase letters, respectively. ${\mathbb{C}}^{N_{ris}\times 1}$ is a complex vector of dimension $N_{ris}$. $diag\left(u\right)$ represents a diagonal matrix whose diagonal elements are the corresponding elements of the vector u. $u^{H}$ denotes the conjugate transpose of the vector u. $Tr\left(U\right)$ and $Rank\left(U\right)$ denote the trace and rank of matrix U, respectively. $CN\left(0,\sigma^{2}\right)$ denotes a complex Gaussian distribution with mean zero and variance $\sigma^{2}$. U≽0 denotes U as a semi-positive definite matrix.

## 2. System Model and Problem Formulation

### 2.1. System Mode

**Figure 1:**
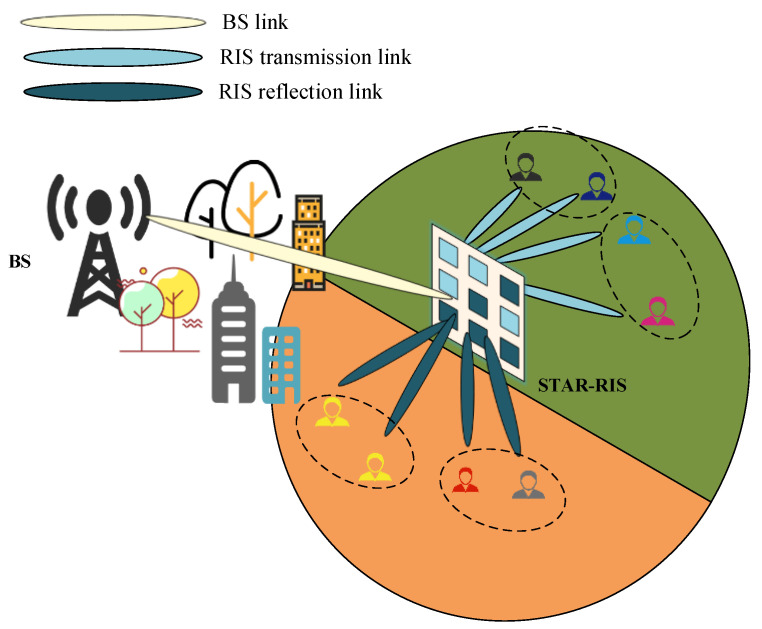
STAR-RIS-assisted NOMA downlink communication system.

As shown in Figure 1, consider the downlink transmission of the STAR-RIS–NOMA system, where the base station (BS) communicates with M single-antenna users, the set of users is $M≜\left\{1,\cdots ,M\right\}$, and the direct link between the users and the BS is blocked. Assume that the BS is equipped with $N_{t}$ transmit antennas, while the STAR-RIS is equipped with $N_{ris}$ elements and the set of elements is $N_{ris}≜\left\{1,\cdots ,N_{ris}\right\}$. All perfect CSIs are available at the BS. $H\in {\mathbb{C}}^{N_{ris}\times N_{t}}$ is the channel from the BS to the STAR-RIS, and $g_{m}\in {\mathbb{C}}^{N_{ris}\times 1}$ is the channel from the STAR-RIS to the m-th user. We use fuzzy C-mean (FCM) clustering to group M users into L clusters. The set of users in the l-th cluster is denoted by $L_{l}$. Thus, the number of users in the l-th cluster can be denoted by $\left|L_{l}\right|$, where $\sum_{l=1}^{L}\left|L_{l}\right|=M$. The detailed process on user clustering will be discussed in Section 3.

This paper considers the user-achievable sum-rate maximization problems under the mode-switching (MS) protocol. Under the MS protocol, STAR-RIS elements are divided into two groups: one working in reflection mode (R mode) and the other in transmission mode (T mode). Let $u^{t}={\left[\sqrt{\beta_{1}^{t}}e^{j\phi_{1}^{t}},\ldots ,\sqrt{\beta_{N_{ris}}^{t}}e^{j\phi_{N_{ris}}^{t}}\right]}^{H}$ and $u^{r}={\left[\sqrt{\beta_{1}^{r}}e^{j\phi_{1}^{r}},\ldots ,\sqrt{\beta_{N_{ris}}^{r}}e^{j\phi_{N_{ris}}^{r}}\right]}^{H}$, where $\beta_{n_{ris}}^{t},\beta_{n_{ris}}^{r}\in \left\{0,1\right\}$,$\beta_{n_{ris}}^{t}+\beta_{n_{ris}}^{r}=1$ and $\phi_{n_{ris}}^{t},\phi_{n_{ris}}^{r}\in [0,2\pi ),\;\forall n_{ris}\in N_{ris}$. The corresponding STAR-RIS transmission and reflection coefficient matrices are $\Phi_{t}=diag\left(u^{t}\right)$ and $\Phi_{r}=diag\left(u^{r}\right)$., respectively.

In the STAR-RIS–NOMA system, $v_{l}$ denotes the active beamforming vector of cluster l, and $s_{l,c}$ denotes the signal sent by the BS to the c-th user of the l-th cluster. Therefore, the received signal of the c-th user of the l-th user cluster is given by

(1) $$\begin{array}{l} y_{l,c}=g_{l,c}^{H}\Phi_{l,c}Hv_{l}\sqrt{\eta_{l,c}}s_{l,c}+\sum_{i\neq c,i\in L_{l}}g_{l,c}^{H}\Phi_{l,c}Hv_{l}\sqrt{\eta_{l,i}}s_{l,i}\\ \;+\sum_{j\neq l,j\in L}\sum_{i\in L_{j}}g_{l,c}^{H}\Phi_{l,c}Hv_{j}\sqrt{\eta_{j,i}}s_{j,i}+n_{l,c}, \end{array}$$

where $\eta_{l,c}$ denotes the power-allocation coefficient of the c-th user of the l-th user -cluster, $n_{l,c}$ represents the additive Gaussian white noise, and $n_{l,c}∼CN\left(0,\sigma^{2}\right)$, $\sigma^{2}$ is the noise power. Due to the presence of multiple users, each user uses the SIC sequentially to eliminate interference. This paper considers the user-achievable sum-rate maximization problem in the case of fixed decoding order. For simplicity and without loss of generality, we assume that the equivalent channel gain order in the l-th user cluster is

(2) $${\left|g_{l,1}^{H}\Phi_{l,1}Hv_{l}\right|}^{2}\geq {\left|g_{l,2}^{H}\Phi_{l,2}Hv_{l}\right|}^{2}\cdots \geq {\left|g_{l,\left|L_{l}\right|}^{H}\Phi_{l,\left|L_{l}\right|}Hv_{l}\right|}^{2}.$$

Thus, the achievable rate for the c-th user of the l-th user cluster can be given as

(3) $$R_{l,c}=\log_{2}\left(1+\frac{{\left|g_{l,c}^{H}\Phi_{l,c}Hv_{l}\right|}^{2}\eta_{l,c}}{{\left|g_{l,c}^{H}\Phi_{l,c}Hv_{l}\right|}^{2}\sum_{i<c}\eta_{l,i}+\sum_{j\neq l,j\in L}\sum_{i\in L_{j}}{\left|g_{l,c}^{H}\Phi_{l,c}Hv_{j}\right|}^{2}\eta_{l,i}+\sigma^{2}}\right).$$

### 2.2. Problem Formulation

To improve the overall data rate, the joint power-allocation coefficients, active beamforming, and transmission and reflection beamforming optimization problems at the RIS are proposed to maximize the achievable rate for users. The problem formulations are

(4a) $$\underset{\eta_{l,c},v_{l},u^{o}}{\max}\sum_{l\in L}\sum_{c\in L_{l}}R_{l,c}$$

(4b) $$s.t.\;R_{l,c}\geq R_{{}_{l,c}}^{\min},$$

(4c) $$\;\sum_{l\in L}{\left\|v_{{}_{l}}\right\|}^{2}\leq P_{\max},$$

(4d) $$\;\phi_{n_{ris}}^{o}\in [0,2\pi ),\;\forall n_{ris}\in N_{ris},$$

(4e) $$\;\beta_{n_{ris}}^{o}\in \left\{0,1\right\},\beta_{n_{ris}}^{t}+\beta_{n_{ris}}^{r}=1,$$

where o∈{t,r}. Constraint (4b) guarantees a minimum quality of service (QoS) requirement for each user, and constraint (4c) indicates a total transmit power budget of $P_{\max}$. Since the objective function is nonconvex, the constraint (4b)–(4d) is also nonconvex. Therefore, problem (4) is a nonconvex optimization problem, which is more difficult to solve directly. Compared with reflection-only RIS, the STAR-RIS has a more complex optimization problem because of the presence of both reflection and transmission modes.

## 3. Channel Feature Extraction and Clustering of Users and STAR-RIS Elements

The number of transmitting antennas arranged at the BS is usually high. The number of STAR-RIS elements is significant, resulting in a high dimensionality of the relevant channels, which is challenging to optimize directly. The Uniform Manifold Approximation and Projection (UMAP) [39] can identify the critical structures in the high-dimensional channel space and preserve these structures in the low-dimensional embedding. Therefore, we first process the channel from the BS to the STAR-RIS and the STAR-RIS to the user using UMAP to extract the critical information and then perform clustering for STAR-RIS elements and system users, respectively.

### 3.1. Channel Feature Extraction Using UMAP

**Figure 2:**
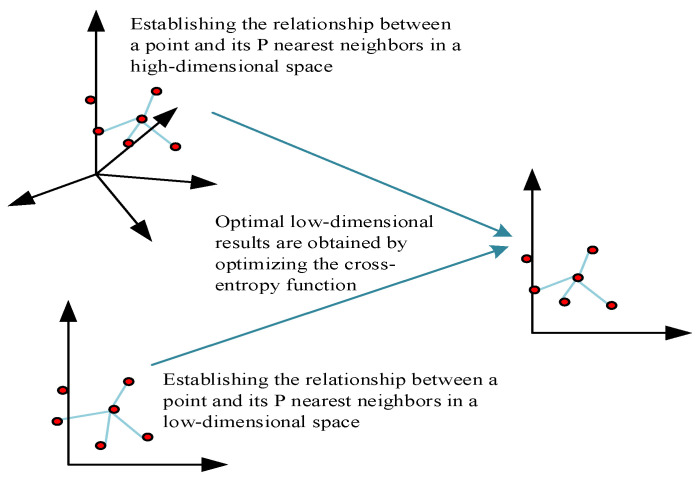
UMAP dimensionality reduction process.

According to the three assumptions of UMAP about the data, a manifold with a fuzzy topology can be modeled. The embedding is found by searching the low-dimensional projection of the data with the closest equivalent fuzzy topology. The process of channel feature extraction using UMAP is shown in Figure 2.

The STAR-RIS-user channel matrix is $G=[g_{1},\ldots ,g_{M}]$. To extract the features of the STAR-RIS-user channel, the first step is to establish the relationship between the high-dimensional samples. In the STAR-RIS-user channel matrix G, the probability-density function between each point can be given as

(5) $$p_{i|j}^{\left(G\right)}=e^{-\frac{d(g_{i}-g_{j})-\rho_{i}^{\left(G\right)}}{\theta_{i}^{\left(G\right)}}},$$

where $d\left(\cdot \right)$ denotes the Euclidean distance between sample points and $\rho_{i}^{\left(G\right)}$ is the distance of the nearest point to point i. To ensure the symmetry of the distance, the joint probability is established as

(6) $$p_{ij}^{\left(G\right)}=p_{i|j}^{\left(G\right)}+p_{j|i}^{\left(G\right)}-p_{i|j}^{\left(G\right)}p_{j|i}^{\left(G\right)}.$$

Fix the $R^{\left(G\right)}$ nearest neighbors of the point i. Solve for $\theta_{i}^{\left(G\right)}$ according to $R^{\left(G\right)}=2^{\sum_{i}p_{ij}^{\left(G\right)}}$. The relationship between the low-dimensional points can be established by Equation (7).

(7) $$q_{ij}^{\left(G\right)}={\left(1+a^{\left(G\right)}{\left({\tilde{g}}_{i}-{\tilde{g}}_{j}\right)}^{b^{\left(G\right)}}\right)}^{-1},$$

where $a^{\left(G\right)}$, $b^{\left(G\right)}$ is the hyperparameter and ${\tilde{g}}_{i}$ is the downscaled channel. In order to describe the relationship between the high-dimensional sample points and the reduced-dimensional sample points, the cross-entropy-loss function is constructed as

(8) $$CE(g_{ij},{\tilde{g}}_{ij})=\sum_{i}\sum_{j}\left[p_{ij}^{\left(G\right)}(g_{ij})\log(\frac{p_{ij}^{\left(G\right)}(g_{ij})}{q_{ij}^{\left(G\right)}({\tilde{g}}_{ij})})+(1-p_{ij}^{\left(G\right)}(g_{ij}))\log(\frac{1-p_{ij}^{\left(G\right)}(g_{ij})}{1-q_{ij}^{\left(G\right)}({\tilde{g}}_{ij})})\right],$$

where $g_{ij}$ denotes the distance between the high-dimensional sample points and ${\tilde{g}}_{ij}$ denotes the distance between the low-dimensional sample points. Therefore, the low-dimensional space ${\tilde{g}}_{i}$, corresponding to $g_{i}$ in the high-dimensional space, can be obtained by solving the following problem:

(9) $$\min\;CE(g_{ij},{\tilde{g}}_{ij})$$

We can use (10) to solve problem (9) using a stochastic gradient descent (SGD) update to obtain the reduced channel matrix $\tilde{G}=\left[{\tilde{g}}_{1},\ldots ,{\tilde{g}}_{M}\right]$.

(10) $${\tilde{g}}_{ij}^{t+1}={\tilde{g}}_{ij}^{t}-\alpha_{1}\nabla_{\tilde{g}}q_{ij}^{\left(G\right)}({\tilde{g}}_{ij}),$$

where $\alpha_{1}$ is the step size.

The channel from the BS to the STAR-RIS is $H={\left[h_{1},\ldots ,h_{N_{ris}}\right]}^{H}$. First, it is necessary to establish the relationship between the high-dimensional samples. The probability-density function between each point in the BS to the STAR-RIS channel matrix H can be given as

(11) $$p_{i|j}^{\left(H\right)}=e^{-\frac{d(h_{i}-h_{j})-\rho_{i}^{\left(H\right)}}{\theta_{i}^{\left(H\right)}}},$$

where $d\left(\cdot \right)$ denotes the Euclidean distance between sample points, and $\rho_{i}^{\left(H\right)}$ is fixed as the distance of the nearest point to point i. To ensure the symmetry of the distance, the joint probability is established:

(12) $$p_{ij}^{\left(H\right)}=p_{i|j}^{\left(H\right)}+p_{j|i}^{\left(H\right)}-p_{i|j}^{\left(H\right)}p_{j|i}^{\left(H\right)}.$$

Fix the $R^{\left(H\right)}$ nearest neighbors of the point i. Solve for $\theta_{i}^{\left(H\right)}$ according to $R^{\left(H\right)}=2^{\sum_{i}p_{ij}^{\left(H\right)}}$. The relationship between the low-dimensional points can be established by (13).

(13) $$q_{ij}^{\left(H\right)}={\left(1+a^{\left(H\right)}{\left({\tilde{h}}_{i}-{\tilde{h}}_{j}\right)}^{b^{\left(H\right)}}\right)}^{-1},$$

where $a^{\left(H\right)}$, $b^{\left(H\right)}$ is the hyperparameter and ${\tilde{h}}_{i}$ is the downscaled channel. To describe the relationship between the high-dimensional sample points and the downscaled low-dimensional sample points, a cross-entropy-loss function is constructed:

(14) $$CE(h_{ij},{\tilde{h}}_{ij})=\sum_{i}\sum_{j}\left[p_{ij}^{\left(H\right)}(h_{ij})\log(\frac{p_{ij}^{\left(H\right)}(h_{ij})}{q_{ij}^{\left(H\right)}({\tilde{h}}_{ij})})+(1-p_{ij}^{\left(H\right)}(h_{ij}))\log(\frac{1-p_{ij}^{\left(H\right)}(h_{ij})}{1-q_{ij}^{\left(H\right)}({\tilde{h}}_{ij})})\right],$$

where $h_{ij}$ denotes the distance between the high-dimensional sample points and ${\tilde{h}}_{ij}$ denotes the distance between the low-dimensional sample points. Therefore, $h_{i}$ in the high-dimensional space corresponds to the low-dimensional space ${\tilde{h}}_{i}$, which can be obtained by solving the following problem:

(15) $$\min\;CE(h_{ij},{\tilde{h}}_{ij})$$

Problem (15) is solved by SGD using (16) to obtain the channel matrix $\tilde{H}={\left[{\tilde{h}}_{1},\ldots ,{\tilde{h}}_{N_{ris}}\right]}^{Τ}$ of the reduced BS to the STAR-RIS.

(16) $${\tilde{h}}_{ij}^{t+1}={\tilde{h}}_{ij}^{t}-\alpha_{2}\nabla_{\tilde{h}}q_{ij}^{\left(H\right)}({\tilde{h}}_{ij}),$$

where $\alpha_{2}$ is the step size.

### 3.2. User Clustering

**Figure 3:**
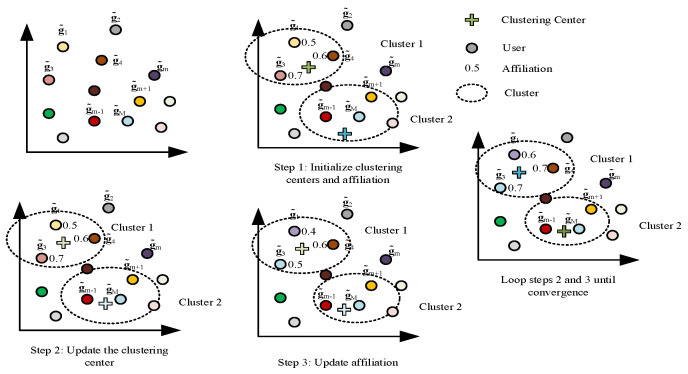
User-clustering process.

After extracting the STAR-RIS-user channel features using UMAP and then using FCM-clustering to subdivide the user clusters, it is possible to classify them more accurately with less complexity. The process of clustering users by FCM is shown in Figure 3. Using FCM, we divide the users into L clusters and the sub-cluster set into $L=\left\{1,\ldots ,L\right\}$. The users are thus classified by the optimization problem (17).

(17a) $$\min\;J_{FCM}^{\left(user\right)}\left(Z,K\right)=\sum_{j=1}^{L}\sum_{i=1}^{M}z_{ji}^{\chi^{\left(user\right)}}{\left\|{\tilde{g}}_{i}-k_{j}\right\|}^{2}$$

(17b) $$s.t.\;\sum_{j=1}^{L}z_{ji}=1,$$

where $\chi^{\left(user\right)}$ is an affiliation factor showing the importance of whether a sample belongs to a class or not, Z is the affiliation matrix $z_{ji}^{\chi^{\left(user\right)}}$ indicating the affiliation of sample i to class j, and $K=\left[k_{1},\ldots ,k_{L}\right]$ is the cluster center. The constraint (17b) is combined into the objective function using the Lagrange multiplier method:

(18) $$J_{FCM}^{\left(user\right)}\left(Z,K,\vartheta_{m}^{\left(user\right)}\right)=\sum_{j=1}^{L}\sum_{i=1}^{M}z_{ji}^{\chi^{\left(user\right)}}{\left\|{\tilde{g}}_{i}-k_{j}\right\|}^{2}+\sum_{m=1}^{M}\vartheta_{m}^{\left(user\right)}\left(\sum_{j=1}^{L}z_{jm}-1\right),$$

where $\vartheta_{m}^{\left(user\right)}$ is the Lagrange multiplier. Taking the derivative of (18) with respect to $z_{ji}$ and $k_{j}$, respectively, yields (19) and (20).

(19) $$\frac{\partial J}{\partial z_{ji}}=\chi^{\left(user\right)}{\left\|{\tilde{g}}_{i}-k_{j}\right\|}^{2}z_{ji}^{\chi^{\left(user\right)}-1}+\vartheta_{i}^{\left(user\right)}=0.$$

(20) $$\frac{\partial J}{\partial k_{j}}=\sum_{i=1}^{M}-2z_{ji}^{\chi^{\left(user\right)}}\left({\tilde{g}}_{i}-k_{j}\right)=0.$$

Solving Equations (19) and (20), we can obtain

(21) $$z_{ji}={\left(\sum_{r=1}^{L}\left(\frac{{\left\|{\tilde{g}}_{i}-k_{j}\right\|}^{\left(\frac{2}{\chi^{\left(user\right)}-1}\right)}}{{\left\|{\tilde{g}}_{i}-k_{r}\right\|}^{\left(\frac{2}{\chi^{\left(user\right)}-1}\right)}}\right)\right)}^{-1}.$$

(22) $$k_{j}=\frac{\sum_{i=1}^{M}\left(z_{ji}^{\chi^{\left(user\right)}}{\tilde{g}}_{i}\right)}{\sum_{i=1}^{M}z_{ji}^{\chi^{\left(user\right)}}}.$$

Equations (21) and (22) are iterated and, finally, the users are divided into L clusters. The set of users in the l-th user cluster is $L_{l}$, and the number of users is $\left|L_{l}\right|$.

### 3.3. STAR-RIS Element Clustering

**Figure 4:**
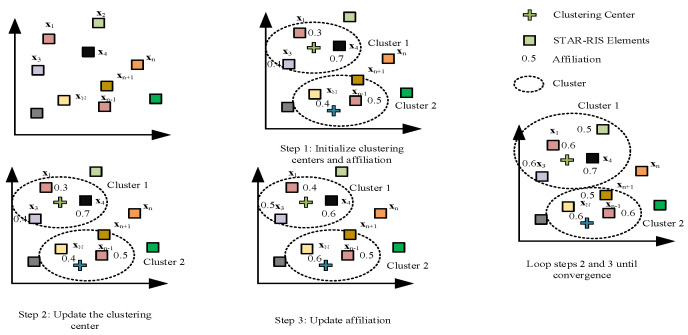
STAR-RIS-element clustering process.

Let $X={\tilde{G}}^{H}{\tilde{H}}^{H}=\left[x_{1},\ldots ,x_{N_{ris}}\right]$; then, also using FCM, divide the elements of the STAR-RIS into $\left|\gamma \right|$ clusters and the set of sub-clusters into $\gamma =\left\{1,\ldots ,\left|\gamma \right|\right\}$. The process of FCM-clustering of STAR-RIS elements is shown in Figure 4, and the classification results of STAR-RIS elements are obtained by the optimization problem (23):

(23a) $$\min\;J_{FCM}^{\left(ris\right)}\left(A,D\right)=\sum_{j=1}^{\left|\gamma \right|}\sum_{i=1}^{N_{ris}}a_{ji}^{\chi^{\left(ris\right)}}{\left\|{\tilde{x}}_{i}-d_{j}\right\|}^{2}$$

(23b) $$s.t.\;\sum_{j=1}^{\left|\gamma \right|}a_{ji}=1,$$

where $\chi^{\left(ris\right)}$ is an affiliation factor showing the importance of whether a sample belongs to a class or not, A is the affiliation matrix, $a_{ji}^{\chi^{\left(ris\right)}}$ indicates the affiliation of sample i belonging to class j, and $D=\left[d_{1},\ldots ,d_{L_{ris}}\right]$ is the cluster center. The constraint (23b) is combined into the objective function using the Lagrange multiplier method:

(24) $$J_{FCM}^{\left(ris\right)}\left(A,D,\vartheta_{m}^{\left(ris\right)}\right)=\sum_{j=1}^{\left|\gamma \right|}\sum_{i=1}^{N_{ris}}a_{ji}^{\chi^{\left(ris\right)}}{\left\|{\tilde{x}}_{i}-d_{j}\right\|}^{2}+\sum_{n=1}^{N_{ris}}\vartheta_{n}^{\left(ris\right)}\left(\sum_{j=1}^{\left|\gamma \right|}a_{jn}-1\right),$$

where $\vartheta_{n}^{\left(ris\right)}$ is the Lagrange multiplier. Equation (24) can be obtained by deriving Equations (25) and (26) for $a_{ji}$ and $d_{j}$ respectively.

(25) $$\frac{\partial J}{\partial a_{ji}}=\chi^{\left(ris\right)}{\left\|{\tilde{x}}_{i}-d_{j}\right\|}^{2}z_{ji}^{\chi^{\left(ris\right)}-1}+\vartheta_{i}^{\left(ris\right)}=0.$$

(26) $$\frac{\partial J}{\partial d_{j}}=\sum_{i=1}^{N_{ris}}-2a_{ji}^{\chi^{\left(ris\right)}}\left({\tilde{x}}_{i}-d_{j}\right)=0.$$

Solving Equations (25) and (26), we obtain

(27) $$a_{ji}={\left(\sum_{r=1}^{\left|\gamma \right|}\left(\frac{{\left\|{\tilde{x}}_{i}-d_{j}\right\|}^{\left(\frac{2}{\chi^{\left(ris\right)}-1}\right)}}{{\left\|{\tilde{x}}_{i}-d_{r}\right\|}^{\left(\frac{2}{\chi^{\left(ris\right)}-1}\right)}}\right)\right)}^{-1}.$$

(28) $$d_{j}=\frac{\sum_{i=1}^{N_{ris}}\left(a_{ji}^{\chi^{\left(ris\right)}}{\tilde{x}}_{i}\right)}{\sum_{i=1}^{N_{ris}}a_{ji}^{\chi^{\left(ris\right)}}}.$$

Equations (27) and (28) are iterated and, finally, the $N_{ris}$ elements of the STAR-RIS are divided into $\left|\gamma \right|$ clusters. The set of elements in the r-th RIS component cluster is $\gamma_{r}$, and the number of elements is $\left|\gamma_{r}\right|$.

After the channel feature extraction and the clustering operation, the received signal of the c-th user of the l-th user cluster transmitted/reflected through the r-th STAR-RIS element cluster is

(29) $$\begin{array}{l} y_{r,l,c}={\tilde{g}}_{r,l,c}^{H}\Phi_{r,l,c}{\tilde{H}}_{r}v_{r,l}\sqrt{\eta_{r,l,c}}s_{r,l,c}+\sum_{i\neq c,i\in L_{l}}{\tilde{g}}_{r,l,c}^{H}\Phi_{r,l,c}{\tilde{H}}_{r}v_{r,l}\sqrt{\eta_{r,l,i}}s_{r,l,i}\\ \;\;\;+\sum_{j\neq l,j\in L}\sum_{i\in L_{j}}{\tilde{g}}_{r,l,c}^{H}\Phi_{r,l,c}{\tilde{H}}_{r}v_{r,j}\sqrt{\eta_{r,j,i}}s_{r,j,i}+n_{r,l,c}. \end{array}$$

We assume that the power-allocation coefficients satisfy $\sum_{c\in L_{l}}\eta_{r,l,c}=1$. Thus, the achievable rate of the c-th user of the l-th user cluster transmitted/reflected through the r-th STAR-RIS cluster elements can be given as Equation (30).

(30) $$R_{r,l,c}=\log_{2}\left(1+\frac{{\left|{\tilde{g}}_{r,l,c}^{H}\Phi_{r,l,c}{\tilde{H}}_{r}v_{r,l}\right|}^{2}\eta_{r,l,c}}{{\left|{\tilde{g}}_{r,l,c}^{H}\Phi_{r,l,c}{\tilde{H}}_{r}v_{r,l}\right|}^{2}\sum_{i<c}\eta_{r,l,i}+\sum_{j\neq l,l\in L}{\left|{\tilde{g}}_{r,l,c}^{H}\Phi_{r,l,c}{\tilde{H}}_{r}v_{r,j}\right|}^{2}+\sigma^{2}}\right).$$

After clustering the STAR-RIS elements and users separately, the STAR-RIS element clusters correspond to the user clusters, in which case $\beta_{n_{ris}}^{t}$ and $\beta_{n_{ris}}^{r}$ are determined. Therefore, to maximize the achievable sum rate of M users, problem (4) is reformulated as

(31a) $$\underset{\eta_{r,l,c},v_{r,l},u^{o}}{\max}\sum_{l\in L}\sum_{c\in L_{l}}R_{r,l,c}$$

(31b) $$s.t.\;R_{{}_{r,l,c}}\geq R_{{}_{r,l,c}}^{\min},$$

(31c) $$\;\sum_{l\in L}{\left\|v_{{}_{r,l}}\right\|}^{2}\leq P_{\max},$$

(31d) $$\phi_{n_{ris}}^{t},\phi_{n_{ris}}^{r}\in [0,2\pi ),\;\forall n_{ris}\in N_{ris},$$

(31e) $$\;\sum_{c\in L_{l}}\eta_{r,l,c}=1,$$

where o∈{t,r}. Let $U_{r.l.c}=u_{r,l,c}u_{r,l,c}^{H}$,$b_{r,l,c}=diag\left({\tilde{g}}_{r,l,c}^{H}\right){\tilde{H}}_{r}$, $B_{r.l.c}=b_{r,l,c}b_{r,l,c}^{H}$ and $V_{r,l}=v_{r,l}v_{r,l}^{H}$, then, the achievable rate is

(32) $$R_{r,l,c}=\log_{2}\left(1+\frac{\eta_{r,l,c}Tr\left(U_{r,l,c}B_{r.l.c}V_{r,l}\right)}{Tr\left(U_{r,l,c}B_{r.l.c}V_{r,l}\right)\sum_{i<c}\eta_{r,l,i}+\sum_{j\neq l,j\in L}Tr\left(U_{r,l,c}B_{r.l.c}V_{r,j}\right)+\sigma^{2}}\right).$$

Problem (31) can be reformulated as

(33a) $$\underset{\eta_{r,l,c},V_{r,l},U_{r,l,c}}{\min}\sum_{l\in L}\sum_{c\in L_{l}}f_{1}\left(\eta_{r,l,c},U_{r,l,c},V_{r,l}\right)+\sum_{l\in L}\sum_{c\in L_{l}}f_{2}\left(\eta_{r,l,c},U_{r,l,c},V_{r,l}\right)$$

(33b) $$s.t.\;f_{3}\left(\eta_{r,l,c},U_{r,l,c},V_{r,l}\right)\leq 0,$$

(33c) $$\;\sum_{l\in L}Tr\left(V_{r,l}\right)\leq P_{\max},$$

(33d) $$\;Rank\left(V_{r,l}\right)=1,$$

(33e) $$\;Rank\left(U_{r,l,c}\right)=1,$$

(33f) $$\;V_{r,l}≽0,$$

(33g) $$\;U_{r,l,c}≽0,$$

(33h) (31e),

where $f_{1}\left(\eta_{r,l,c},U_{r,l,c},V_{r,l}\right)$ is as in (34), $f_{2}\left(\eta_{r,l,c},U_{r,l,c},V_{r,l}\right)$ is as in Equation (35), and $f_{3}\left(\eta_{r,l,c},U_{r,l,c},V_{r,l}\right)$ is as in Equation (36). Problem (33) is still nonconvex, and we use SDR to relax the rank-one constraint and then use a first-order Taylor expansion to approximate the nonconvex part of the objective function with the constraint present.

(34) $$f_{1}\left(\eta_{r,l,c},U_{r,l,c},V_{r,l}\right)=\log_{2}\left(Tr\left(U_{r,l,c}B_{r.l.c}V_{r,l}\right)\sum_{i<c}\eta_{r,l,i}+\sum_{j\neq l,j\in L}Tr\left(U_{r,l,c}B_{r.l.c}V_{r,j}\right)+\sigma^{2}\right).$$

(35) $$f_{2}\left(\eta_{r,l,c},U_{r,l,c},V_{r,l}\right)=-\log_{2}\left(Tr\left(U_{r,l,c}B_{r.l.c}V_{r,l}\right)\sum_{i\leq c}\eta_{r,l,i}+\sum_{j\neq l,j\in L}Tr\left(U_{r,l,c}B_{r.l.c}V_{r,j}\right)+\sigma^{2}\right).$$

(36) $$\begin{array}{l} f_{3}\left(\eta_{r,l,c},U_{r,l,c},V_{r,l}\right)=\left(Tr\left(U_{r,l,c}B_{r.l.c}V_{r,l}\right)\sum_{i<c}\eta_{r,l,i}+\sum_{j\neq l,j\in L}Tr\left(U_{r,l,c}B_{r.l.c}V_{r,j}\right)+\sigma^{2}\right)\left(2^{R_{{}_{r,l,c}}^{\min}}-1\right).\\ \;-\eta_{r,l,c}Tr\left(U_{r,l,c}B_{r.l.c}V_{r,l}\right) \end{array}$$

## 4. Solution of the Problem

In this section, we propose a new alternating optimization iterative algorithm to solve the joint optimization problem of power-allocation coefficients, active beamforming, and STAR-RIS passive beamforming.

### 4.1. Power-Allocation Coefficient Optimization

When fixing the STAR-RIS coefficients and the BS beamforming vectors, problem (33) can be reformulated as

(37a) $$\underset{\eta_{r,l,c}}{\min}\sum_{l\in L}\sum_{c\in L_{l}}f_{1}\left(\eta_{r,l,c}\right)+\sum_{l\in L}\sum_{c\in L_{l}}f_{2}\left(\eta_{r,l,c}\right)$$

(37b) $$s.t.\;f_{3}\left(\eta_{r,l,c}\right)\leq 0,$$

(37c) (31e).

At this point, the constraint is convex, and the first-order Taylor expansion of $f_{1}\left(\eta_{r,l,c}\right)$ is

(38) $$f_{1}\left(\eta_{r,l,c}\right)\geq f_{1}\left(\eta_{r,l,c}^{\left(\tau \right)}\right)+Tr\left({\left(\nabla_{\eta }f_{1}\left(\eta_{r,l,c}^{\left(\tau \right)}\right)\right)}^{H}\left(\eta_{r,l,c}-\eta_{r,l,c}^{\left(\tau \right)}\right)\right)≜f_{1}\left(\eta_{r,l,c},\eta_{r,l,c}^{\left(\tau \right)}\right),$$

where $\eta_{r,l,c}^{\left(\tau \right)}$ denotes the value of $\eta_{r,l,c}$ at the τ-th iteration and, according to the SCA principle, Equation (37a) can be replaced by

(39) $$\underset{\eta_{r,l,c}}{\min}\sum_{l\in L}\sum_{c\in L_{l}}f_{1}\left(\eta_{r,l,c},\eta_{r,l,c}^{\left(\tau \right)}\right)+\sum_{l\in L}\sum_{c\in L_{l}}f_{2}\left(\eta_{r,l,c}\right)$$

Using the penalty-function method, problem (37) can be transformed into an unconstrained optimization problem:

(40) $$\underset{\eta_{r,l,c}}{\min}\sum_{l\in L}\sum_{c\in L_{l}}f_{1}\left(\eta_{r,l,c},\eta_{r,l,c}^{\left(\tau \right)}\right)+\sum_{l\in L}\sum_{c\in L_{l}}f_{2}\left(\eta_{r,l,c}\right)+\zeta_{1}\left(\sum_{c\in L_{l}}\eta_{r,l,c}^{2}-1+\max{\left\{0,f_{3}\left(\eta_{r,l,c}\right)\right\}}^{2}\right)$$

where $\zeta_{1}$ is the penalty factor. Thus, the gradient-descent method can be used to solve for $\eta_{r,l,c}$ by using Equation (41).

(41) $$\begin{array}{l} \eta_{r,l,c}^{\left(\tau +1\right)}=\eta_{r,l,c}^{\left(\tau \right)}-\alpha_{3}\left(\sum_{l\in L}\sum_{c\in L_{l}}\nabla_{\eta }f_{1}^{\left(\tau \right)}\left(\eta_{r,l,c},\eta_{r,l,c}^{\left(\tau -1\right)}\right)\right)+\alpha_{3}\left(\sum_{l\in L}\sum_{c\in L_{l}}\nabla_{\eta }f_{2}^{\left(\tau \right)}\left(\eta_{r,l,c}\right)\right)\\ \;\;\;\;\;+\alpha_{3}\zeta_{1}\left(2\sum_{c\in L_{l}}\eta_{r,l,c}^{\left(\tau \right)}+\nabla_{\eta }^{\left(\tau \right)}\left(\max{\left\{0,f_{3}\left(\eta_{r,l,c}\right)\right\}}^{2}\right)\right), \end{array}$$

where $\alpha_{3}$ is the step size.

### 4.2. Active Beamforming Optimization

Fixing the STAR-RIS coefficients and power-allocation coefficients, problem (33) can be formulated as

(42a) $$\underset{V_{r,l}}{\min}\sum_{l\in L}\sum_{c\in L_{l}}f_{1}\left(V_{r,l}\right)+\sum_{l\in L}\sum_{c\in L_{l}}f_{2}\left(V_{r,l}\right)$$

(42b) $$s.t.\;f_{3}\left(V_{r,l}\right)\leq 0,$$

(42c) (33c),(33d),(33f).

Using the SDR relaxation rank-one constraint (33d), the first-order Taylor expansion of $f_{1}\left(V_{r,l}\right)$ is

(43) $$f_{1}\left(V_{r,l}\right)\geq f_{1}\left(V_{r,l}^{\left(\tau \right)}\right)+Tr\left({\left(\nabla_{V}f_{1}\left(V_{r,l}^{\left(\tau \right)}\right)\right)}^{H}\left(V_{r,l}-V_{r,l}^{\left(\tau \right)}\right)\right)\;≜f_{1}\left(V_{r,l},V_{r,l}^{\left(\tau \right)}\right),$$

where $V_{r,l}^{\left(\tau \right)}$ denotes the value of $V_{r,l}$ at the τ-th iteration. Thus, the objective function (42a) can be converted to

(44) $$\underset{V_{r,l}}{\min}\sum_{l\in L}\sum_{c\in L_{l}}f_{1}\left(V_{r,l},V_{r,l}^{\left(\tau \right)}\right)+\sum_{l\in L}\sum_{c\in L_{l}}f_{2}\left(V_{r,l}\right)$$

After removing the constraints (42b) and (33c) using the penalty function, problem (42) can be converted to

(45a) $$\underset{V_{r,l}}{\min}\sum_{l\in L}\sum_{c\in L_{l}}f_{1}\left(V_{r,l},V_{r,l}^{\left(\tau \right)}\right)+\sum_{l\in L}\sum_{c\in L_{l}}f_{2}\left(V_{r,l}\right)+\zeta_{2}\left({\left[\max\left\{0,f_{3}\left(V_{r,l}\right),\sum_{l\in L}Tr\left(V_{r,l}\right)-P_{\max}\right\}\right]}^{2}\right)$$

(45b) s.t. (33f),

Problem (45) is a semidefinite programming problem (SDP), which is solved by using the BCD [40] method. First, problem (45) is converted into an unconstrained optimization problem:

(46) $$\begin{array}{l} \underset{V_{r,l}}{\min}\sum_{l\in L}\sum_{c\in L_{l}}f_{1}\left(V_{r,l},V_{r,l}^{\left(\tau \right)}\right)+\sum_{l\in L}\sum_{c\in L_{l}}f_{2}\left(V_{r,l}\right)\\ \;+\zeta_{2}\left({\left[\max\left\{0,f_{3}\left(V_{r,l}\right),\sum_{l\in L}Tr\left(V_{r,l}\right)-P_{\max}\right\}\right]}^{2}\right)-\varpi_{1}\log\det\left(V_{r,l}\right) \end{array}$$

where $\varpi_{1}>0$ is the barrier factor. The key to applying the coordinate-descent method to the problem of (46) is the following block determinant:

(47) $$\det\left(V_{r,l}\right)=\det\left(S\right)\det\left(\overset{⌢}{y}-{\overset{⌢}{x}}^{T}S^{-1}\overset{⌢}{x}\right),$$

where $V_{r,l}=\left(\begin{array}{cc} S & \overset{⌢}{x}\\ {\overset{⌢}{x}}^{T} & \overset{⌢}{y} \end{array}\right)$, and therefore, problem (46) can be transformed by solving

(48) $$\begin{array}{l} \underset{\overset{⌢}{x}}{\min}\sum_{l\in L}\sum_{c\in L_{l}}{\overset{⌢}{f}}_{1}\left(\overset{⌢}{x}\right)+\sum_{l\in L}\sum_{c\in L_{l}}{\overset{⌢}{f}}_{2}\left(\overset{⌢}{x}\right)\\ \;+\zeta_{2}\left({\left[\max\left\{0,{\overset{⌢}{f}}_{3}\left(\overset{⌢}{x}\right),\sum_{l\in L}C_{l}^{T}\overset{⌢}{x}-P_{\max}\right\}\right]}^{2}\right)-\varpi_{1}\log\left(\overset{⌢}{y}-{\overset{⌢}{x}}^{T}S^{-1}\overset{⌢}{x}\right) \end{array}$$

where ${\overset{⌢}{f}}_{1}\left(\overset{⌢}{x}\right)$ is (49), ${\overset{⌢}{f}}_{2}\left(\overset{⌢}{x}\right)$ is (50), and ${\overset{⌢}{f}}_{3}\left(\overset{⌢}{x}\right)$ is Equation (51).

(49) $${\overset{⌢}{f}}_{1}\left(\overset{⌢}{x}\right)=\log_{2}\left(w_{l,c}^{T}\overset{⌢}{x}\sum_{i<c}\eta_{r,l,i}+\sum_{j\neq l,j\in L}w_{l,j}^{T}\overset{⌢}{x}+\sigma^{2}\right).$$

(50) $${\overset{⌢}{f}}_{2}\left(\overset{⌢}{x}\right)=-\log_{2}\left(w_{l,c}^{T}\overset{⌢}{x}\sum_{i\leq c}\eta_{r,l,i}+\sum_{j\neq l,j\in L}w_{l,j}^{T}\overset{⌢}{x}+\sigma^{2}\right).$$

(51) $${\overset{⌢}{f}}_{3}\left(\overset{⌢}{x}\right)=\left(w_{l,c}^{T}\overset{⌢}{x}\sum_{i<c}\eta_{r,l,i}+\sum_{j\neq l,j\in L}w_{l,j}^{T}\overset{⌢}{x}+\sigma^{2}\right)R_{{}_{r,l,c}}^{MIN}-\eta_{r,l,c}w_{l,c}^{T}\overset{⌢}{x},$$

where $R_{{}_{r,l,c}}^{MIN}=2^{R_{{}_{r,l,c}}^{\min}}-1$.

The gradient-descent method is used to solve for $\overset{⌢}{x}$ according to Equation (52).

(52) $$\begin{array}{l} {\overset{⌢}{x}}^{\left(\tau +1\right)}={\overset{⌢}{x}}^{\left(\tau \right)}-\alpha_{4}\left(\begin{array}{l} \sum_{l\in L}\sum_{c\in L_{l}}\nabla_{\overset{⌢}{x}}{\overset{⌢}{f}}_{1}^{\left(\tau \right)}\left(\overset{⌢}{x},{\overset{⌢}{x}}^{\left(\tau -1\right)}\right)+\sum_{l\in L}\sum_{c\in L_{l}}\nabla_{\overset{⌢}{x}}{\overset{⌢}{f}}_{2}^{\left(\tau \right)}\left(\overset{⌢}{x}\right)\\ \; \end{array}\right)\\ \; \end{array}\;\begin{array}{l} \;\;\;\;+\alpha_{4}\zeta_{2}\left(\nabla_{\overset{⌢}{x}}^{\left(\tau \right)}{\left[\max\left\{0,{\overset{⌢}{f}}_{3}\left(\overset{⌢}{x}\right),\sum_{l\in L}w_{l}^{T}\overset{⌢}{x}-P_{\max}\right\}\right]}^{2}\right)+\alpha_{4}\frac{2\varpi_{1}}{\overset{⌢}{y}-{\overset{⌢}{x}}^{T}S^{-1}\overset{⌢}{x}}S^{-1}\overset{⌢}{x}, \end{array}$$

where $\alpha_{4}$ is the step size.

### 4.3. STAR-RIS Coefficient Optimization

Fixing the power-allocation coefficients and BS beamforming vectors, problem (33) can be formulated as

(53a) $$\underset{U_{r,l,c}}{\min}\sum_{l\in L}\sum_{c\in L_{l}}f_{1}\left(U_{r,l,c}\right)+\sum_{l\in L}\sum_{c\in L_{l}}f_{2}\left(U_{r,l,c}\right)$$

(53b) $$s.t.\;f_{3}\left(U_{r,l,c}\right)\leq 0,$$

(53c) (33e),(33g).

Regarding the relaxation of constraint (33e) using SDR, the first-order Taylor expansion of $f_{1}\left(U_{r,l,c}\right)$ is

(54) $$f_{1}\left(U_{r,l,c}\right)\geq f_{1}\left(U_{r,l,c}^{\left(\tau \right)}\right)+Tr\left(\left({\left(\nabla_{U}f_{1}\left(U_{r,l,c}^{\left(\tau \right)}\right)\right)}^{H}\left(U_{r,l,c}-U_{r,l,c}^{\left(\tau \right)}\right)\right)\right)≜f_{1}\left(U_{r,l,c},U_{r,l,c}^{\left(\tau \right)}\right),$$

where $U_{r,l,c}^{\left(\tau \right)}$ denotes the value of $U_{r,l,c}$ at the τ-th iteration. The objective function (53a) can be re-expressed as

(55) $$\underset{U_{r,l,c}}{\min}\sum_{l\in L}\sum_{c\in L_{l}}f_{1}\left(U_{r,l,c},U_{r,l,c}^{\left(\tau \right)}\right)+\sum_{l\in L}\sum_{c\in L_{l}}f_{2}\left(U_{r,l,c}\right)$$

Convert problem (53) to problem (56) using the penalty function.

(56a) $$\underset{U_{r,l,c}}{\min}\sum_{l\in L}\sum_{c\in L_{l}}f_{1}\left(U_{r,l,c},U_{r,l,c}^{\left(\tau \right)}\right)+\sum_{l\in L}\sum_{c\in L_{l}}f_{2}\left(U_{r,l,c}\right)+\zeta_{3}\left({\left[\max\left\{0,f_{3}\left(U_{r,l,c}\right)\right\}\right]}^{2}\right)$$

(56b) s.t. (33g),

where $\zeta_{3}$ is the penalty factor. Problem (56) is an SDP problem, and the solution using the BCD method can convert (56) into an unconstrained problem.

(57) $$\begin{array}{l} \underset{U_{r,l,c}}{\min}\sum_{l\in L}\sum_{c\in L_{l}}f_{1}\left(U_{r,l,c},U_{r,l,c}^{\left(\tau \right)}\right)+\sum_{l\in L}\sum_{c\in L_{l}}f_{2}\left(U_{r,l,c}\right)\\ \;\;\;\;+\zeta_{3}\left({\left[\max\left\{0,f_{3}\left(U_{r,l,c}\right)\right\}\right]}^{2}\right)-\varpi_{2}\log\det\left(U_{r,l,c}\right) \end{array}$$

where $\varpi_{2}>0$ is the barrier factor. The key to applying the coordinate-descent method to the problem of (57) is the following block determinant:

(58) $$\det\left(U_{r,l,c}\right)=\det\left(O\right)\det\left(\hat{y}-{\hat{x}}^{T}O^{-1}\hat{x}\right),$$

where $U_{r,l,c}=\left(\begin{array}{cc} O & \hat{x}\\ {\hat{x}}^{T} & \hat{y} \end{array}\right)$; therefore, problem (57) can be transformed by solving

(59) $$\underset{\hat{x}}{\min}\sum_{l\in L}\sum_{c\in L_{l}}{\hat{f}}_{1}\left(\hat{x}\right)+\sum_{l\in L}\sum_{c\in L_{l}}{\hat{f}}_{2}\left(\hat{x}\right)+\zeta_{3}\left({\left[\max\left\{0,{\hat{f}}_{3}\left(\hat{x}\right)\right\}\right]}^{2}\right)-\varpi_{2}\log\left(\hat{y}-{\hat{x}}^{T}O^{-1}\hat{x}\right)$$

where ${\hat{f}}_{1}\left(\hat{x}\right)$ is shown in Equation (60), ${\hat{f}}_{2}\left(\hat{x}\right)$ is shown in (61), and ${\hat{f}}_{3}\left(\hat{x}\right)$ is shown in Equation (62).

(60) $${\hat{f}}_{1}\left(\hat{x}\right)=\log_{2}\left({\hat{w}}_{l,c}^{T}\hat{x}\sum_{i<c}\eta_{r,l,i}+\sum_{j\neq l,j\in L}{\hat{w}}_{l,j}^{T}\hat{x}+\sigma^{2}\right).$$

(61) $${\hat{f}}_{2}\left(\hat{x}\right)=-\log_{2}\left({\hat{w}}_{l,c}^{T}\hat{x}\sum_{i\leq c}\eta_{r,l,i}+\sum_{j\neq l,j\in L}{\hat{w}}_{l,j}^{T}\hat{x}+\sigma^{2}\right).$$

(62) $${\hat{f}}_{3}\left(\hat{x}\right)=\left({\hat{w}}_{l,c}^{T}\hat{x}\sum_{i<c}\eta_{r,l,i}+\sum_{j\neq l,j\in L}{\hat{w}}_{l,j}^{T}\hat{x}+\sigma^{2}\right)R_{{}_{r,l,c}}^{MIN}-\eta_{r,l,c}{\hat{w}}_{l,c}^{T}\hat{x}.$$

The gradient-descent method is used to solve for $\hat{x}$ according to Equation (63).

(63) $$\begin{array}{l} {\hat{x}}^{\left(\tau +1\right)}={\hat{x}}^{\left(\tau \right)}-\alpha_{5}\left(\sum_{l\in L}\sum_{c\in L_{l}}\nabla_{\hat{x}}{\hat{f}}_{1}^{\left(\tau \right)}\left(\hat{x},{\hat{x}}^{\left(\tau -1\right)}\right)+\sum_{l\in L}\sum_{c\in L_{l}}\nabla_{\hat{x}}{\hat{f}}_{2}^{\left(\tau \right)}\left(\hat{x}\right)\;\right)\\ \;\;\;\;\;\;\;\;+\alpha_{5}\zeta_{3}\left(\nabla_{\hat{x}}^{\left(\tau \right)}{\left[\max\left\{0,{\hat{f}}_{3}\left(\hat{x}\right)\right\}\right]}^{2}\right)+\frac{2\alpha_{5}\varpi_{2}}{\hat{y}-{\hat{x}}^{T}O^{-1}\hat{x}}O^{-1}\hat{x}, \end{array}$$

where $\alpha_{5}$ is the step size factor.

In Algorithm 1, the complexity of computing the power allocation is $O_{1}≜O\left(\tau_{1}^{\max}M^{3.5}\log_{2}\left(\frac{1}{\varepsilon_{1}}\right)\right)$, where $\tau_{1}^{\max}$ is the number of iterations of Algorithm 1, and $\varepsilon_{1}$ denotes the accuracy of Algorithm 1. In Algorithm 2, the complexity of the active beam formation is calculated as $O_{2}≜O\left(\tau_{2}^{\max}N_{t}\log\left(N_{t}\right)\right)$, where $\tau_{2}^{\max}$ is the number of iterations of the algorithm. In Algorithm 3, the complexity of calculating the STAR-RIS passive beam formation is $O_{3}≜O\left(\tau_{3}^{\max}\left|\gamma_{r}\right|\log\left(\left|\gamma_{r}\right|\right)\right)$, where $\tau_{3}^{\max}$ is the number of iterations of the algorithm. Thus, the total complexity of the algorithm is $O\left(\tau_{4}^{\max}\left(O_{1}+O_{2}+O_{3}\right)\right)$, where $\tau_{4}^{\max}$ denotes the number of outer iterations.
**Algorithm 1:** Proposed Penalty-Based Iterative Algorithm for Solving Problem (37)1: Initialize feasible point $\eta_{r,l,c}^{\left(0\right)}$$,\;\mathrm{and}\;\mathrm{penalty}\;\mathrm{factor}\;\zeta_{1}$.2: **repeat:**3:  Set the iteration index τ=0.4:  **repeat:**5:  $\mathrm{Use}\;f_{1}\left(\eta_{r,l,c},\eta_{r,l,c}^{\left(\tau \right)}\right)$$\;\mathrm{to}\;\mathrm{approximate}\;f_{1}\left(\eta_{r,l,c}\right)$.6:  $\mathrm{Update}\;\eta_{r,l,c}^{\left(\tau +1\right)}$ by solving problem (40) using (41).7:  τ=τ+1.8:    **until** the fraction of objective function values decreases below a predefined threshold or the maximum number of internal iterations is reached.9: $\mathrm{Update}\;\eta_{r,l,c}^{\left(0\right)}$.10:  $\mathrm{Update}\;\mathrm{penalty}\;\mathrm{factor}\;\zeta_{1}$.11: **until** the constraint violation is below a predefined threshold.

**Algorithm 2:** Proposed Penalty-Based Iterative Algorithm for Solving Problem (42)1: Initialize the feasible point a $V_{r,l}^{\left(0\right)}$$,\;\mathrm{penalty}\;\mathrm{factor}\;\zeta_{2}$.2: **repeat:**3: Set the iteration index τ=0.4:  **repeat:**5:  $\mathrm{For}\;a\;\mathrm{given}\;V_{r,l}^{\left(\tau \right)}$$,\;\mathrm{set}\;\mathrm{the}\;\mathrm{barrier}\;\mathrm{factor}\;\varpi_{1}$, and use BCD to convert the solution of problem (46) to problem (48).6:  $\mathrm{Update}\;{\overset{⌢}{x}}^{\left(\tau +1\right)}$ by (52).7:  $\mathrm{Update}\;V_{r,l}^{\left(\tau +1\right)}$ with the obtained optimal solution.8:  τ=τ+1.9:  **until** the fraction of objective function values decreases below a predefined threshold or the maximum number of internal iterations is reached.$10:\;\mathrm{Update}\;V_{r,l}^{\left(0\right)}$.12: **until** the constraint violation is below a predefined threshold.

**Algorithm 3:** Proposed Penalty-Based Iterative Algorithm for Solving Problem (53)1: Initialize feasible point $U_{r,l,c}^{\left(0\right)}$$,\;\mathrm{and}\;\mathrm{penalty}\;\mathrm{factor}\;\zeta_{3}$.2: **repeat:**3: Set the iteration index τ=0.4:  **repeat:**5:  $\mathrm{For}\;a\;\mathrm{given}\;U_{r,l,c}^{\left(\tau \right)}$$,\;\mathrm{set}\;\mathrm{the}\;\mathrm{barrier}\;\mathrm{factor}\;\varpi_{2}$ and convert problem (57) to problem (59).6:  $\mathrm{Update}\;{\hat{x}}^{\left(\tau +1\right)}$ using (63).7:  $\mathrm{Update}\;U_{r,l,c}^{\left(\tau +1\right)}$ with the obtained optimal solution.8:  τ=τ+1.9:  **until** the fraction of objective function values decreases below a predefined threshold or the maximum number of internal iterations is reached.$10:\;\mathrm{Update}\;U_{r,l,c}^{\left(0\right)}$.12: **until** the constraint violation is below a predefined threshold.

## 5. Numerical Results

**Figure 5:**
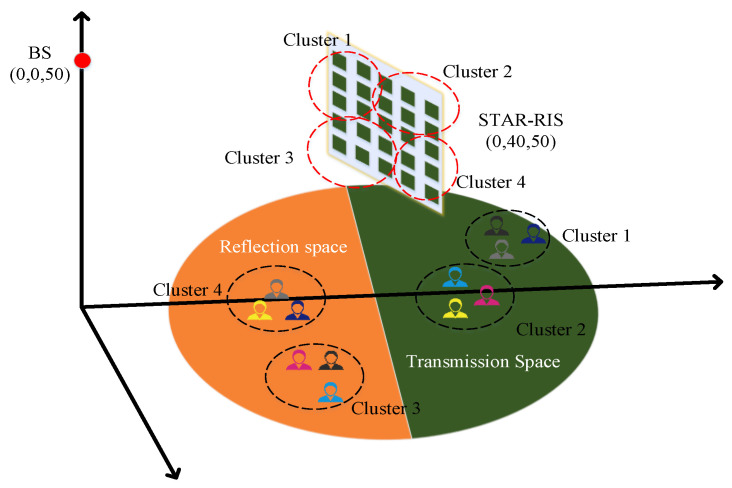
STAR-RIS simulation scenario.

In this section, numerical simulations are performed to evaluate the performance of the proposed algorithm. The specific simulation scenario is shown in Figure 5. Without loss of generality, it is assumed that the BS and the STAR-RIS are located at (0, 0, 50) and (0, 40, 50), respectively. The STAR-RIS–NOMA system has M=12 users. The users are divided into L=4 clusters with cluster center coordinates of (0, 20, 0), (0, 30, 15), (0, 50, 0), and (0, 60, −15), respectively, and the users are randomly distributed in clusters corresponding to a radius of 6. It is assumed that STAR-RIS is equipped with $N_{ris}=N_{RIS}^{h}N_{ris}^{v}$ elements, where $N_{RIS}^{h}=5$ denotes the number of elements along the horizontal direction, $N_{RIS}^{v}=5$ represents the number of elements along the vertical path, and STAR-RIS elements are divided into $\left|\gamma \right|=4$ clusters. The narrowband quasi-static fading channel from the BS to the STAR-RIS and from the STAR-RIS to the user is modeled as a Rician-fading channel as follows:

(64) $$H=\sqrt{\frac{\varsigma_{0}}{\xi_{BR}^{\omega_{BR}}}}\left(\frac{B_{BR}}{B_{BR}+1}H^{Los}+\sqrt{\frac{1}{B_{BR}+1}}H^{NLos}\right),$$

(65) $$g_{l,c}=\sqrt{\frac{\varsigma_{0}}{\xi_{RU,l,c}^{\omega_{RU}}}}\left(\frac{B_{RU}}{B_{RU}+1}g_{l,c}^{Los}+\sqrt{\frac{1}{B_{RU}+1}}g_{l,c}^{NLos}\right),$$

where $\xi_{BR}$ and $\xi_{RU,l,c}$ denote the distance between the BS and the STAR-RIS and the distance between the STAR-RIS and the user, respectively; $\varsigma_{o}$ denotes the path loss at a reference distance of 1 m; $\omega_{BR}$ and $\omega_{RU}$ denote the corresponding path loss index; $B_{BR}$ and $B_{RU}$ denote the Rician factors; and $H^{Los}$ and $g_{l,c}^{Los}$ are the determined line-of-sight (LoS) components. $H^{NLos}$ and $g_{l,c}^{NLos}$ are the random non-line-of-sight (NLoS) components modeled using Rician fading. In order not to lose generality, assume that users have the same QoS requirements and set $R_{l,c}^{\min}=0.1\;\mathrm{bits}∕s∕\mathrm{Hz}$. The system parameters used are shown in Table 1.

**Figure 6:**
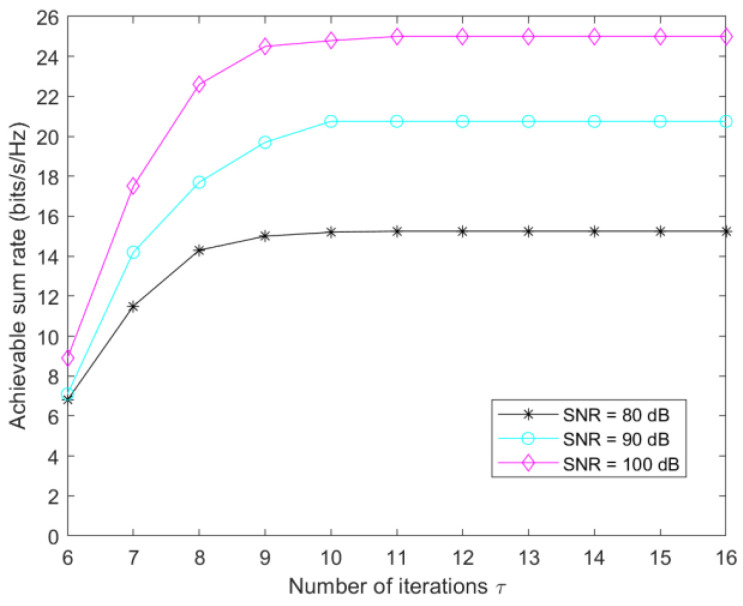
Convergence of the power-allocation algorithm.

Figure 6 investigates the convergence of the power-allocation algorithm. With $P_{\max}=35\mathrm{dBm}$, $N_{ris}=25$, and $N_{t}=5$, the user-achievable sum rate of the algorithm converges in about 11 iterations as the number of iterations increases.

**Figure 7:**
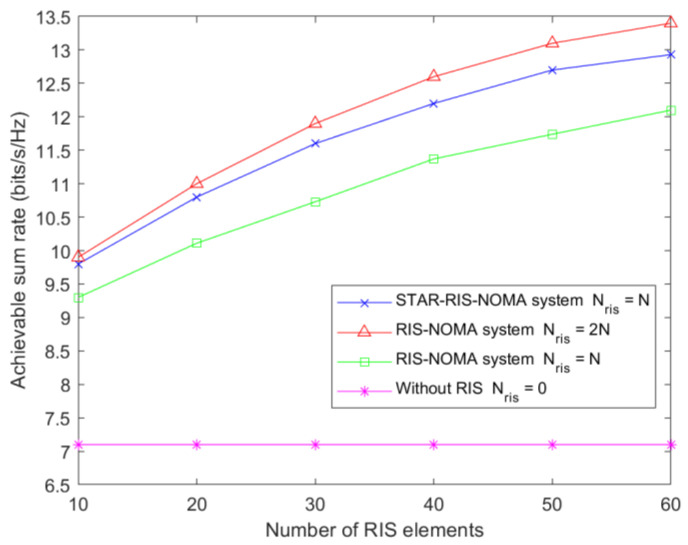
Achievable sum rate relative to the number of RIS elements for $P_{\max}=35\;\mathrm{dBm}$ and $N_{t}=5$.

Figure 7 shows the relationship between the user-achievable sum rate and the number of RIS elements. A total of four cases are considered. The first case is the use of the STAR-RIS in the NOMA system, where the number of elements of STAR-RIS is $N_{ris}=N$. In the second case, two conventional RISs are used in the system: one for reflection and the other for transmission, with N elements per RIS. The third case also uses conventional RISs, but each RIS has $\frac{N}{2}$ elements. The fourth type is the NOMA system without RIS assistance. The results show that the performance of the STAR-RIS is higher than that of the conventional RIS for the same number of RIS elements. The higher version of the dual RIS than the STAR-RIS is because the dual RIS has N reflected and N transmitted elements at the same moment, while the transmitted/reflected elements of the STAR-RIS add up to a total of N.

**Figure 8:**
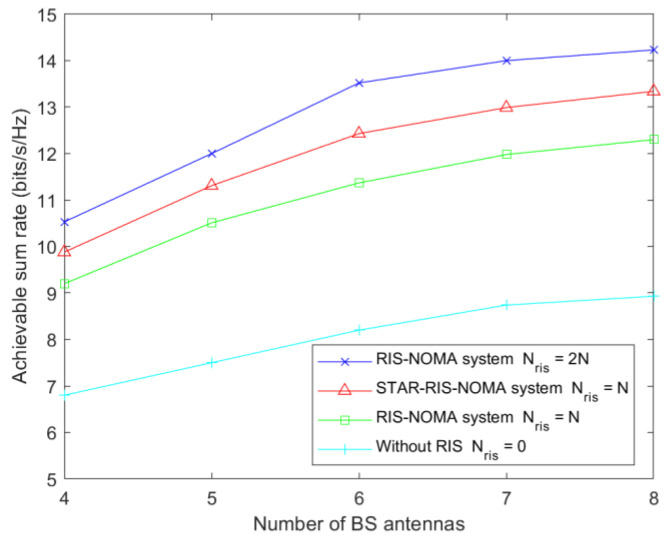
Achievable sum rate relative to the number of BS elements for $P_{\max}=35\;\mathrm{dBm}$ and $N_{ris}=25$.

Figure 8 shows the user-achievable sum rate versus the BS transmit antenna in the case of conventional RIS-assisted NOMA and STAR-RIS-assisted NOMA communications. The experimental results show that the use of STAR-RIS-assisted communication is superior to a conventional RIS when the number of BS antennas is the same, given the same number of RIS elements. It can be observed that using RIS in NOMA can significantly improve the system’s performance. Furthermore, as the number of antennas at the BS increases, the performance of the RIS–NOMA system also increases.

**Figure 9:**
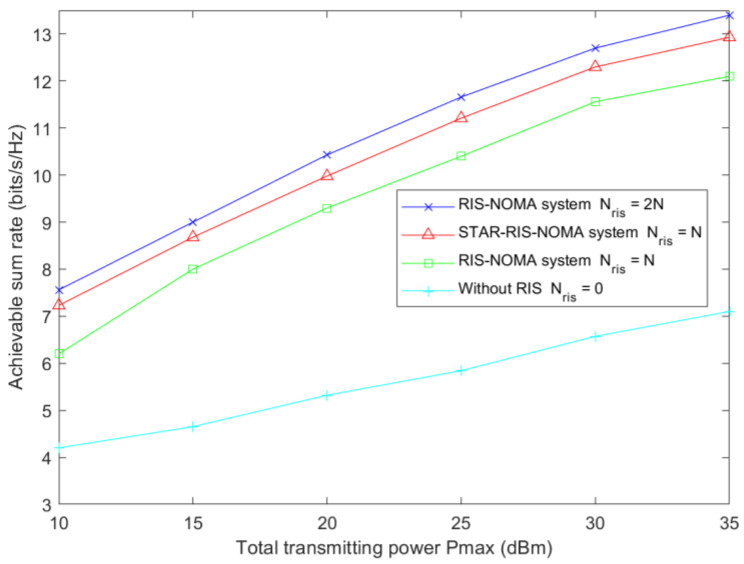
Achievable sum rate relative to total transmitting power for $N_{ris}=25$ and $N_{t}=5$.

Figure 9 shows the user-achievable sum rate versus the total transmitted power Pmax during conventional RIS and STAR-RIS-assisted NOMA communications. The user-achievable sum rate increases with the total transmission power. RIS-assisted communications significantly improve user achievability and rates over NOMA systems without RIS assistance. It was observed that the performance difference between the system using two conventional RISs and the STAR-RIS system was relatively small.

**Figure 10:**
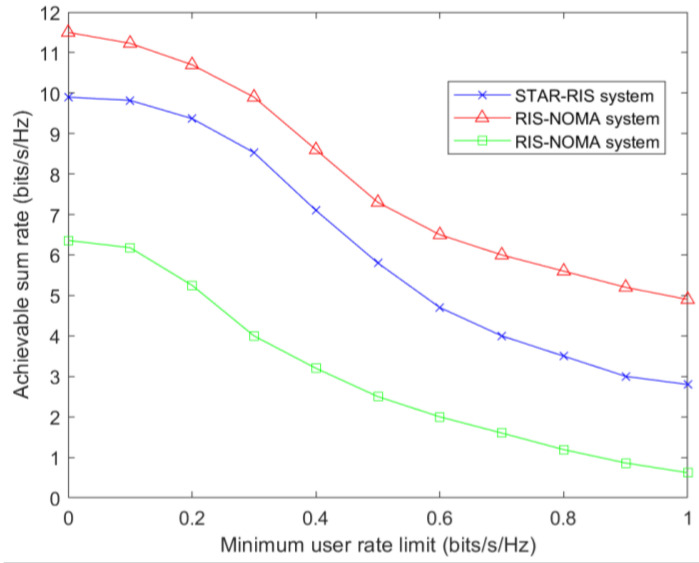
Achievable sum rate versus minimum user rate for $P_{\max}=35\;\mathrm{dBm}$, $N_{ris}=25$ and $N_{t}=5$.

Figure 10 considers the relationship between the user-achievable sum rate and the minimum user rate for three cases: a STAR-RIS system with N elements, a RIS system with 2N elements, and a RIS system with N elements. The results show that the user-achievable sum rate decreases as the minimum user rate increases. In all three cases, the RIS system with 2N components has the highest user-achievable sum rate, and the STAR-RIS system with N elements performs better than the RIS system with N elements.

**Figure 11:**
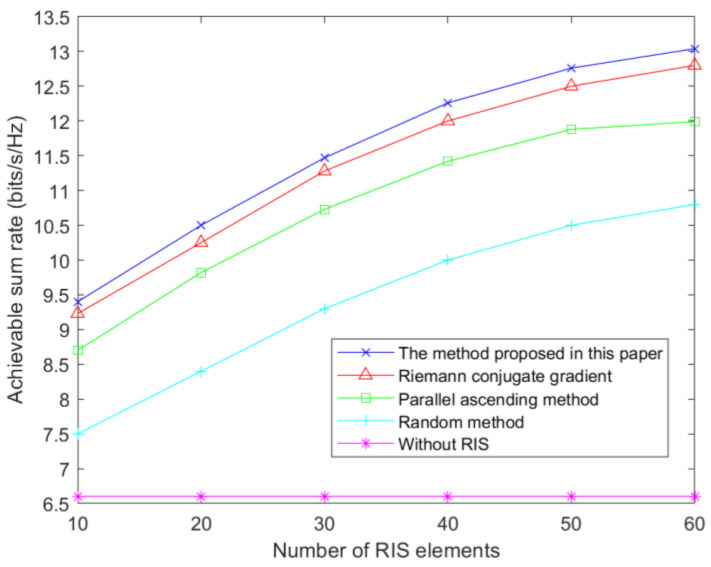
Achievable sum rate relative to the number of RIS elements under different methods.

Figure 11 shows how the proposed method in this paper compares with four other methods or schemes. The first scheme is where no RIS is configured in the system. The second scheme is where each cell of the STAR-RIS is randomly configured with a phase. The third scheme uses a pairwise-rising method [41] to determine the phase shift of the STAR-RIS. The fourth scheme is to use the Riemann conjugate-gradient method [42] to solve the phase-shift matrix of the STAR-RIS. As can be seen from the results in Figure 11, when the number of RIS elements is 60, the user-achievable sum rate of the proposed method in this paper is 13.1 bits/s/Hz. The achievable sum rate of the random phase-shift method is only 10.8 bits/s/Hz. The achievable sum rates using the pairwise-ascent method and the Riemann conjugate-gradient method are 12.8 bits/s/Hz and 12 bits/s/Hz, respectively.

**Figure 12:**
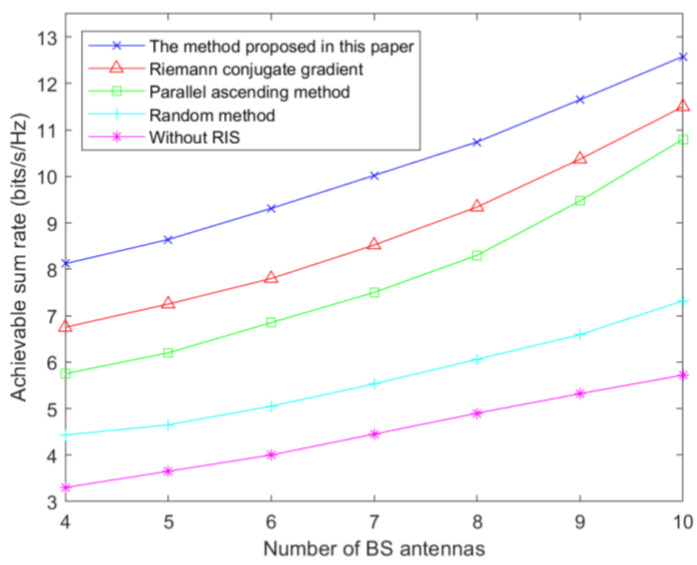
Achievable sum rate relative to the number of BS antennas under different methods.

Figure 12 shows the variation of achievable users and rates with the number of antennas at the BS. When the number of BS transmitting antennas is 10, the user-achievable sum rate of the random phase shift is 7.3 bits/s/Hz. The user-achievable sum rates of the pairwise-ascent method and Riemann conjugate-gradient method are 10.8 bits/s/Hz and 11.5 bits/s/Hz, respectively. The achievable sum rate of the proposed method is 12.6 bits/s/Hz. Among the five schemes, the worst performance is achieved without RIS assistance, followed by the random RIS phase shift. The Riemann conjugate-gradient method is closer to the case of the pairwise-ascent method, and the proposed method in this paper has the best performance. Therefore, this shows the effectiveness of the proposed algorithm.

## 6. Conclusions

In this paper, we study the downlink transmission system of the STAR-RIS–NOMA. A new alternate optimization method is proposed for the MS protocol to jointly optimize the power-allocation coefficient of the user, active beamforming at the BS, and passive beamforming of the STAR-RIS so that the achievable rate of the user is maximized. The channel is first feature-extracted, and the user and STAR-RIS elements are clustered according to the extracted features to reduce the solution difficulty. The SDR technique and penalty function are used to solve the nonconvex factors present in the problem. The effectiveness of the proposed algorithm can be seen in the simulation results.

This paper considers the STAR-RIS in an ideal state, where the transmission and reflection phase-shift coefficients can be adjusted independently. However, in real applications, coupled transmission and reflection phase-shift coefficients are more common, and the related research problems are more complex. Therefore, the design of coupled STAR-RIS phase-shift coefficients is a crucial research direction for the future.

## Figures and Tables

**Table 1:** System Parameters.

Parameter	Parameter Explanation	Value
$B_{BR}$	Rician factor for BS to STAR-RIS channels	3 dB
$B_{RU}$	Rician factor of STAR-RIS to user channel	3 dB
$\varsigma_{o}$	Path loss at a reference distance of 1 m	−30 dB
$\omega_{BR}$	Path loss index for BS to STAR-RIS channels	2.2
$\omega_{RU}$	Path Loss Index for Path Loss Index	2.2
$\zeta_{1},\zeta_{2},\zeta_{3},\zeta_{4}$	Initialize penalty factor	10^−4^
$\tau_{\max}$	Maximum number of inner-layer iterations	25

## Data Availability

Not applicable.
